# Organizational knowledge translation strategies for allied health professionals in traumatology settings: realist review protocol

**DOI:** 10.1186/s13643-021-01793-4

**Published:** 2021-09-23

**Authors:** Karine Latulippe, Annie LeBlanc, Marie-Pierre Gagnon, Katia Boivin, Pascale Lavoie, Joëlle Dufour, Emmanuelle Paquette Raynard, Eve Richard, Marie-Ève Lamontagne

**Affiliations:** 1 Centre de recherche interdisciplinaire en réadaptation du Montréal métropolitain (CRIR), Pavillon Lindsay de l’IURDPM, 6363, Chemin Hudson, Montréal, Québec H3S 1M9 Canada; 2 Département de médecine familiale et de médecine d’urgence, Faculté de médecine, Pavillon Ferdinand-Vandry, Université Laval, 1050 av. de la Médecine, Québec, Québec G1V 0A6 Canada; 3 Chaire de recherche du Canada en technologies et pratiques en santé, Faculté des sciences infirmières, Pavillon Ferdinand-Vandry, Université Laval, Canada, 1050 av. de la Médecine, Québec, Québec G1V 0A6 Canada; 4 Direction de l’enseignement et des affaires universitaires, CHU de Québec-Université Laval, 2705 Boulevard Laurier, Québec, Québec G1V 4G2 Canada; 5 Direction de l’enseignement et des affaires universitaires, Centre intégré universitaire de santé et de services sociaux de la Capitale-Nationale, 525, boul. Wilfrid Hamel, Québec, Québec G1M 2S8 Canada; 6 Centre interdisciplinaire de recherche en réadaptation et intégration sociale, Institut de réadaptation en déficience physique de Québec, 525 boul. Wilfrid-Hamel, Québec, Québec G1M 2S8 Canada; 7 Bibliothèque de l’Université Laval, 2345, allée des Bibliothèques, Québec, Québec G1V 0A6 Canada

**Keywords:** Knowledge translation, Knowledge mobilization, Knowledge management, Transfer of learning, Training management, Organizational strategies, Evidence-based practices, Realist review

## Abstract

**Background:**

Knowledge translation (KT) is an important means of improving the health service quality. Most research on the effectiveness of KT strategies has focused on individual strategies, i.e., those directly targeting the modification of allied health professionals’ knowledge, attitudes, and behaviors, for example. In general, these strategies are moderately effective in changing practices (maximum 10% change). Effecting change in organizational contexts (e.g., change readiness, general and specific organizational capacity, organizational routines) is part of a promising new avenue to service quality improvement through the implementation of evidence-based practices. The objective of this study will be to identify why, how, and under what conditions organizational KT strategies have been shown to be effective or ineffective in changing the (a) knowledge, (b) attitudes, and (c) clinical behaviors of allied health professionals in traumatology settings.

**Methods:**

This is a realist review protocol involving four iterative steps: (1) Initial theory formulation, (2) search for Evidence search, (3) knowledge extraction and synthesis, and (4) recommendations. We will search electronic databases such as PubMed, Embase, CINHAL, Cochrane Library, and Conference Proceedings Citation Index - Science. The studies included will be those relating to the use of organizational KT strategies in trauma settings, regardless of study designs, published between January 1990 and October 2020, and presenting objective measures that demonstrate change in allied health professionals’ knowledge, attitudes, and clinical behaviors. Two independent reviewers will select, screen, and extract the data related to all relevant sources in order to refine or refute the context-mechanism-outcome (CMO) configurations developed in the initial theory and identify new CMO configurations.

**Discussion:**

Using a systematic and rigorous method, this review will help guide decision-makers and researchers in choosing the best organizational strategies to optimize the implementation of evidence-based practices.

**Systematic review registration:**

PROSPERO CRD42020216105

**Supplementary Information:**

The online version contains supplementary material available at 10.1186/s13643-021-01793-4.

## Background

Knowledge translation (KT) [1] is an important means of improving health service quality [2]. The science of KT is currently booming [1]. However, most research to date has focused on the upstream stages of the process (e.g., assessing facilitators and barriers or monitoring the use of interventions), and only a limited number of studies have examined the effectiveness of KT strategies [3, 4]. KT effectiveness refers to the ability of the different strategies employed to produce results for users, stakeholders, and the health system [3, 4]. Research on KT strategy effectiveness has primarily dealt with individual strategies, i.e., strategies that directly target the modification of health workers’ knowledge, attitudes, and behaviors [5, 6]. In general, these strategies are moderately effective in changing practices (maximum 10% change) [5]. In this context, it is imperative to consider other KT avenues than those focused on individuals alone in order to continue to improve health service quality.

A promising new avenue for supporting service quality improvement through the translation of evidence-based practices targets changes in organizational contexts (e.g., change readiness, general and specific organizational capacity, organizational routines) [7–11]. Increased emphasis [12–14] is being put on the decisive influence that organizational KT strategies could have on evidence-based practice implementation. This influence is now included in the majority of theoretical and conceptual models explaining this phenomenon [15–17]. Organizational strategies are defined by Wensing et al. [5] as “planned rearrangements of one or more aspects of the organization of patient care.” In contrast to individual strategies, organizational KT strategies focus on the changes in the care and service contexts of organizations, such as redefining professional roles, changing meeting structures, and care protocols. These strategies, in addition to those focused on the individual, have the potential to significantly catalyze health and social service quality improvement. However, their mechanisms of action and effectiveness have been poorly investigated and remain unclear [18]. Recent studies conducted in the Quebec trauma rehabilitation context have highlighted the value of organizational implementation strategies to promote the effective and sustainable implementation of evidence-based practices [19–22]. For example, changes in disciplinary roles, interprofessional meeting structures, and physical changes to the work environment were reported as facilitating implementation in this particular context. In 2008, Wensing et al. conducted a structured review of systematic reviews of the effectiveness of organizational strategies to improve professional practices and health outcomes for users; their results were equivocal, as none of the documented strategies produced consistent results. In addition, only the effectiveness of these strategies was analyzed, without any attempt to open the “black box” of their functioning. The action mechanisms of these strategies have not been documented, and it is therefore difficult to understand how they may or may not contribute to changing the behaviors of health professionals. Furthermore, in 2012, Flogren et al. conducted a systematic review of the effectiveness of organizational structures in promoting the use of evidence-based practice in nursing; they found only one study with a sufficiently rigorous design that did not conclude on the effectiveness of the strategy used for 3 months post-implementation [23]. As regards our rehabilitation practice context specifically, it is characterized by certain particularities in terms of KT due to the interdisciplinary nature of interventions, on the one hand, and the evolutionary nature of patient pathologies and hence their impact on patients’ capacity and lifestyle, on the other.

A realist review is required to assess the different action mechanisms of organizational KT strategies (how, why, for whom, to what extent, and under what circumstances) and to facilitate the choice of effective strategies that will enable effective and sustainable implementation of evidence-based practice for allied health professionals in traumatology settings. Realist synthesis (or review) is a scientific literature review approach that provides an explanatory analysis of how theories work (or fail to work) in different contexts [24]. It is used to develop an understanding of the implementation chain of interventions by detailing the prerequisites to achieving positive results and avoiding negative outcomes [24]. No research has been done to date on the functioning and effectiveness of organizational KT strategies with allied health professionals in traumatology settings. Focusing on a new organizational target can improve KT effectiveness and service quality. The objective of this study will be to identify why, how, and under what conditions organizational KT strategies have been shown to be effective or ineffective in changing the (a) knowledge, (b) attitudes, and (c) clinical behaviors of allied health professionals in traumatology settings.

## Methods

### Study design

We will conduct a realist review [24]. We report this realist review protocol following the Preferred Reporting Items for Systematic Reviews and Meta-Analyses for Protocols (PRISMA-P) statement [25] (see checklist in Additional file 1). This protocol has been registered within the International Prospective Register of Systematic Reviews (PROSPERO) database (registration ID: CRD42020216105). The final review will be reported in accordance with the Realist And Meta-narrative Evidence Syntheses: Evolving Standards (RAMESES) guideline [26].

### Research team

The research team consists of seven co-investigators: three academic researchers (MEL, AL, MPG), two knowledge users (KB, PL), one research coordinator (JD), and one postdoctoral fellow (KL). The project involves a multidisciplinary team of experts in knowledge synthesis (AL), neurotrauma rehabilitation (MEL), management in traumatology settings (KB, PL), and KT (MEL, AL, MPG). Knowledge users are decision-makers representing health and social services settings in Quebec City. The postdoctoral fellow is working on developing expertise in knowledge synthesis and KT. All team members will contribute to the different stages of this project through document revision, reflection, and decision-making with regard to the methodological approach and the various analyses. Monthly written follow-up is planned, as well as two face-to-face meetings at the beginning of the project and one at the end of the project. Other meetings (by telephone or in-person) may be convened as needed in the course of the project.

### Stages of the realist review

The realist review consists of four main stages: (1) formulation of initial theories, (2) search for evidence, (3) knowledge extraction and synthesis, and (4) recommendations [24].

#### Formulation of initial theories

In terms specific to the realist review, we will put forward initial theories to explain the context-mechanism-outcome (CMO) system. From a methodological point of view, researchers begin by extracting from the literature the main ideas that relate to a class of interventions (the initial theories). These initial theories explain how and why a class of interventions (in this case, organizational KT strategies) work and generate the outcome(s) of interest (in this case, change in the knowledge, attitudes, and clinical behaviors of allied health professionals in traumatology settings).

We will use the Consolidated Framework for Implementation Research (CFIR) [15] to guide the creation of the initial theories. The CFIR is a metamodel that groups constructs used in 19 different KT theories. This model distinguishes five main areas of factors influencing the achievement of KT strategies: (1) the characteristics of the evidence-based practices to be implemented, (2) the external context, (3) the internal context, (4) the individuals involved, and (5) the implementation techniques. CFIR is widely used in the study of complex implementation projects where interactions between factor categories are expected [27].

Based on CFIR and team members’ own clinical and organizational experience, we will develop initial theories explaining why, how, and in what context organizational KT strategies can work. Specifically, we will hold a 1.5-h, audio-recorded, group discussion to develop a first draft theory answering the following question: how (context and mechanism) an organizational KT strategy (intervention) has been shown to be effective or ineffective in changing the (a) knowledge, (b) attitudes, and (c) clinical behaviors of allied health professionals (outcomes). We will make an initial schematic representation based on the discussion and refine it through revision of the audio-recorded material. This representation will then be tested against data from studies included in the review.

#### Evidence search and eligibility criteria

##### Information source and search strategy

The search will be conducted in the MEDLINE, CINAHL, Embase, Cochrane, Ergonomic Abstracts, and Web of Science Core Collection databases and may be extended to other databases of a more social or sociological nature as required (planned date coverage between January 1990 and October 2020). It should be noted that, depending on the realist review methodology, subsequent iterative research may be conducted to generate additional data on a particular aspect of the research question.

Two knowledge management specialists will perform the primary article search in the various databases. Subsequently, the research team will use three different mechanisms: (1) the list of cited references will be reviewed to extract articles of interest, (2) the “find similar articles” function of the databases will be used to check if other articles of interest are related to the primary articles, and (3) the authors who have published more than two articles deemed relevant will be contacted by e-mail to request a list of relevant references, published or not, on our subject. All articles found during the secondary search will in turn be put through the three secondary search mechanisms to complete the search. Finally, Grey Matters, a practical tool for searching health-related grey literature [28], will be used to complement the secondary research. The list of articles will be recorded in a specialized reference management software (Endnote) and checked to eliminate duplicates. The search will include a broad range of terms and keywords related to knowledge translation + trauma + allied health professionals. A full draft search strategy for MEDLINE (OVID) is provided in Additional file 2.

##### Eligibility criteria

We will consider French- and English-language studies relating to organizational KT strategies published. The definition of KT used for the purpose of article selection  is the one provided by the Canadian Institutes of Health Research (https://cihr-irsc.gc.ca/e/29529.html):

A dynamic and iterative process that includes synthesis, dissemination, exchange, and ethically sound application of knowledge to improve the health of Canadians provide more effective health services and products and strengthen the health care system.

We believe that the last 30 years are sufficient to identify organizational KT strategies that correspond to the reality of the current health network. All study designs will be considered. We will include studies conducted with allied health professionals (occupational therapists, physiotherapists, social workers, psychologists, speech-language pathologists, sexologists) in traumatology settings. For the outcomes, we will include all objective measures that demonstrate a change in the clinical knowledge, attitudes, and behaviors of allied health professionals.

#### Selection, extraction, and knowledge synthesis

##### Study selection

First (by title and abstract) and second (full-text review) screenings will be carried out independently by two reviewers (KL, MEL) using Covidence [29]. We will pilot the screening form across a random sample of 20 titles and abstracts. In the event of a difference of opinion for a given article, a third researcher will take a position on the eligibility of the study. The same method will be applied to full-text reviews. The appraisal of the articles will focus on (a) relevance, i.e., does the study create at least one CMO configuration about organizational KT strategies, and (b) rigor, i.e., whether this CMO configuration has sufficient weight to make a methodologically credible contribution [24]. This will lead to the development of the final list of included articles that will be retained for the data collection process. It is possible that the choice of articles may raise questions about the focus of the review and, as a result, iteratively reflect on the research question and the selection of articles [24]. These questions and reflections will be put to the research team.

##### Data collection process

We will carry out a thorough reading of the selected articles followed by the extraction of relevant data. The extraction will be also performed in parallel by two research team members (MEL, JD) using a standard Covidence [29] extraction form to reduce variability and bias [30]. Categories of data extraction will include the following:

- Study characteristics: title, first author, publication year, country, participants (which allied health professional), study objectives, trauma setting (hospital, community), and outcomes (what specific changes have been measured in the (a) knowledge, (b) attitudes, and (c) clinical behaviors)
- Intervention: type of organizational KT strategies
- Program theory: refined or refuted CMO configurations developed in the initial theory and new CMO configurations
- Methodology: data collection method and type of analysis

The extraction form will be piloted with five studies. Here again, data extraction can lead to questions about the focus of the synthesis and, consequently, to iterative reflection on the research question and selection of articles. These questions and reflections will also be referred to the research team.

#### Data/knowledge synthesis and recommendations

Two independent analysts will examine the selected articles and indicate to what extent they correspond to each CMO configurations developed in the original theory. They will also identify any new CMOs that may emerge from the studies. Results will then be compare in a cross-tabulation matrix. A consensus will have to be reached before results are presented to the rest of the team. Data synthesis will take into account the circumstances in which these organizational KT strategies were used, including the characteristics of the targeted evidence-based practices, as well as internal, external, and personal contexts of individuals involved, in addition to any other information that supports the understanding of the context. Based on the emerging results of the studies, the research team will be able to validate (confirm or refute) the initial theories on the effectiveness of organizational KT strategies through group discussion. The team will perform a comparative analysis between the initial theories and the emerging results of the review as well as a search for both contradictory and consensual results [24, 26]. Specifically, they will meet for a 1.5-h, audio-recorded group meeting. From this discussion, several proposals for CMO combinations will emerge to better specify which organizational KT strategies are most effective in influencing professionals’ knowledge, attitudes, and behaviors. According to the data, it will be possible to identify patterns of CMO and to identify a final theory that will enable a theoretical generalization of the observed results in other contexts [31]. This in turn will “[p]rovide guidance on what policy-makers or practitioners could put in place to change the context or provide resources in a way that most likely triggers the appropriate mechanism(s) to produce the desired outcome” [26]. Together with the knowledge users on the research team we will determine based on the final results how to best present our findings to their colleagues. Traditional methods such as article publication and  presentation at national or international conferences will also be used.

## Discussion

Using a systematic and rigorous method, this review will help guide decision-makers and researchers in choosing the best organizational strategies to optimize the implementation of evidence-based practices in the trauma field.

Researchers have explained some of the challenges that are specific to realist reviews. Among other things, making the distinction between mechanism and context can be difficult [32–34]. Robert and Ride explain that the mechanism (M) in one CMO configuration can be the context (C) of another CMO configuration [34]. Moreover, Pawson et al. mention there is a limit to what can be understood through the available information. Less tangible information such as relationships of influence and power, for example, may not be described in an article [35], hence the importance of opening up the search for information to documents other than primary studies, but this also makes it more difficult to assess the quality of the information. In this context, quality assessment is not necessarily based on formal grids, but mainly on the judgment of the authors of the synthesis with regard to data credibility and its impact on theories [35]. The recommendations based on the synthesis may also be limited. It will be difficult for the authors of the synthesis to claim that they have grasped all the opportunities and constraints associated with interventions and to predict the circumstances under which subsequent projects will be implemented [35]. This limits the scope of the recommendations. It is crucial that future users of the knowledge produced through realist synthesis understand the usefulness, but also the limitations of this approach. This underscores the importance of their participation in the review from the outset. Finally, it is important to remember that the steps described in this protocol will be carried out in an iterative manner. Any amendments made to this protocol when conducting the study will be outlined and reported in the final manuscript.

## Supplementary Information

**Additional file 1.** PRISMA-P populated checklist.

**Additional file 2.** Draft search strategy for MEDLINE.

## Data Availability

Not applicable.

## Abbreviations

CFIR: Consolidated Framework for Implementation Research
CMO: Context-mechanism-outcome
KT: Knowledge translation
RAMESES: Realist And Meta-narrative Evidence Syntheses: Evolving Standards
