# Supplementary material for: Organizational knowledge translation strategies for allied health professionals in traumatology settings: realist review protocol
Source: Syst Rev. 2021 Sep 23;10:255. doi: 10.1186/s13643-021-01793-4 (PMC8461924; doi:10.1186/s13643-021-01793-4)
Supplement: Supplementary file 2 — Additional file 2. Draft search strategy for MEDLINE. [file 13643_2021_1793_MOESM2_ESM.docx]

| **Medline (OVID) V2** | | | |
| --- | --- | --- | --- |
| # | Requête | 06-oct |  |
| 1 | Translational Medical Research/ | 10996 |  |
| 2 | ("translational medical research*" or "Translational Medical Science*" or "translational medicine" or "translational research*").ab,kf,ti. | 13481 |  |
| 3 | exp Evidence-Based Practice/ | 88831 |  |
| 4 | exp Knowledge Management/ | 362 |  |
| 5 | exp "Diffusion of Innovation"/ | 20060 |  |
| 6 | exp Health Knowledge, Attitudes, Practice/ | 112496 |  |
| 7 | ((knowledge* or Research* or innovation* or science*) adj5 (transfer* or translat* or broker* or mobili#ation or adapt* or implement* or exchange* or application or utili#ation or communicat* or cycle or transform* or action or populari* or adopt* or dissemination or sharing or diffusion or uptake)).ti,ab,kf. | 122300 |  |
| 8 | ((evidence or research or knowledge or theory) adj5 (practice or policy) adj5 gap).ti,ab,kf. | 2350 |  |
| 9 | "know-do".ti,ab,kf. | 114 |  |
| 10 | or/1-9 | 334909 |  |
| 11 | Accidental Injuries/ or Amputation, Traumatic/ or Ankle Injuries/ or Anterior Cruciate Ligament Injuries/ or Arm Injuries/ or Back Injuries/ or Blast Injuries/ or Brain Concussion/ or Brain Contusion/ or Brain Injuries, Traumatic/ or Brain Injuries/ or Burns/ or Burns, Chemical/ or Burns, Electric/ or Central Cord Syndrome/ or Chronic Traumatic Encephalopathy/ or Clavicle injuries/ or Contusions/ or Corneal Injuries/ or Crush Injuries/ or Cumulative Trauma Disorders/ or exp Joint Dislocations/ or Eye Injuries/ or Facial Injuries/ or Finger Injuries/ or Forearm Injuries/ or exp Fractures, Bone/ or Hand Injuries/ or Head Injuries, Penetrating/ or Head Injuries, Closed/ or Hip Injuries/ or Knee Injuries/ or Leg Injuries/ or Maxillofacial Injuries/ or Neck Injuries/ or Occupational Injuries/ or Post-Concussion Syndrome/ or Shock, Traumatic/ or Spinal Cord Compression/ or Spinal Cord Injuries/ or Spinal Injuries/ or Whiplash Injuries/ or "Wounds and Injuries"/ or Wounds, Gunshot/ or Wounds, Stab/ or Wrist Injuries/ | 552961 |  |
| 12 | ("ACL Tear*" or "Acromioclavicular Joint* Separation*" or "Ankle Sprain*" or "Anterior Cruciate Ligament Tear*" or "Bankart Lesion*" or "Bankart Tear*" or "Battle* Sign*" or "Bone* Stress* Reaction*" or "Brain Concussion*" or "Brain Concussion*" or "Brain Contusion*" or "Brain Laceration*" or "Burn*" or "Central Cord Injury Syndrome" or "Central Cord Syndrome" or "Central Spinal Cord Syndrome" or "Cerebellar Contusion*" or "Cerebral Concussion*" or "Cerebral Contusion*" or "Chronic Post Concussive Encephalopath*" or "Chronic Post Traumatic Encephalopath*" or "Chronic Post-Concussive Encephalopath*" or "Chronic Post-Traumatic Encephalopath*" or "Chronic Traumatic Encephalopath*" or "Commotio Cerebri" or "Compressive Myelopathy" or "Contusio Cerebri" or "contusion*" or "Conus Medullaris Syndrome*" or "Corneal Abrasion*" or "Corneal Damage*" or "Corneal Scar*" or "Cortical Contusion*" or "Diastas*" or "Dislocation*" or "Fracture* " or "Glenohumeral Subluxation*" or "Growth Plate Injur*" or "Hill-Sachs Defect*" or "Hill-Sachs Lesion*" or "Hip* Displacement*" or "Hip* Dysplasia" or "injur*" or "Intermediate Concussion*" or "Joint Subluxation*" or "Luxatio Erecta" or "Mild Concussion*" or "Myodiastas*" or "Overuse Syndrome*" or "Post Concussive Encephalopath*" or "Post Concussive Symptom*" or "Post Concussive Syndrome*" or "Post Traumatic Encephalopath*" or "Postconcussion Syndrome*" or "Post-Concussion Syndrome*" or "Post-Concussive Encephalopath*" or "Post-Traumatic Encephalopath*" or "Repetitive Motion Disorder*" or "Severe Concussion*" or "Spinal Cord* Compression*" or "Spinal Cord* Contusion*" or "Spinal Cord* Laceration*" or "Spinal Cord* Transection*" or "TBI*" or "trauma*" or "Traumatic Encephalopath*" or "traumatic shock*" or "Traumatic* Amputation*" or "Turf toe*" or "wound*").ti,ab. | 1511990 |  |
| 13 | 11 or 12 | 1639177 |  |
| 14 | exp Occupational Therapists/ | 324 |  |
| 15 | exp Physical Therapists/ | 1948 |  |
| 16 | exp Allied Health Personnel/ | 49911 |  |
| 17 | ("occupational therap*" or "physical therapist*" or physiotherapist* or Ergotherap* or kinesiologist* or paramedic* or "medical technician*" or "home care aide*" or "home health aide*" or sexolog* or psycholog* or "social worker*" or "speech-language pathologist*").ab,kf,ti. | 342103 |  |
| 18 | ("allied health" adj3 (personnel or occupation* or staff or profession*)).ab,kf,ti. | 3208 |  |
| 19 | ((healthcare or "health care") adj2 assistant*).ab,kf,ti. | 857 |  |
| 20 | ("community health" adj2 (worker* or aide*)).ab,kf,ti. | 4809 |  |
| 21 | (psychiatric adj3 (aide* or orderl* or attendant*)).ab,kf,ti. | 127 |  |
| 22 | ((physician* or doctor* or medical* or nurse*) adj3 assistant*).ab,kf,ti. | 4922 |  |
| 23 | exp rehabilitation/ | 307262 |  |
| 24 | Rehabilitation Centers/ | 8218 |  |
| 25 | (pharmac* adj2 (aide* or technician*)).ab,kf,ti. | 1139 |  |
| 26 | (neurorehabilitation* or rehabilitation*).ab,kf,ti. | 174044 |  |
| 27 | ("support personnel" or "support worker*" or (communit* adj2 organization*)).ab,kf,ti. | 6522 |  |
| 28 | (audiolog* OR telerehabilitation* or (dental adj2 (auxiliar* or assistant* or technician*) or denturist*)).ab,kf,ti. | 14875 |  |
| 29 | or/14-28 | 801994 |  |
| 30 | 10 and 13 and 29 | 2261 |  |
| 31 | avec dans concept 3 (nurse* or nursing)ti,ab,kf. Ligne donne 1217976 | 3378 |  |
